# Branch retinal vein occlusion with sectoral cystoid macular edema in toxoplasmic chorioretinitis

**DOI:** 10.11604/pamj.2013.16.118.3182

**Published:** 2013-11-26

**Authors:** Zouheir Hafidi, Rajae Daoudi

**Affiliations:** 1 Université Mohammed V Souissi, Service d'Ophtalmologie A de l'hôpital des spécialités, Centre Hospitalier Universitaire, Rabat, Maroc

**Keywords:** Retinal vein occlusion, macular edema, chorioretinitis, toxoplasmosis

## Image in medicine

A 28 year-old man presented with sudden visual loss in his left eye. Best-corrected visual acuity was 20/20 in the right eye and 20/400 in the left eye. Fundus examination showed a whitish retinal lesion of the superotemporal quadrant involving the supero-temporal retinal vein branch with mild vitritis. Fluorescein angiography confirmed active retinochoroidal inflammation (black arrow) with delayed filling and tortuosity of the involved vein (white arrow); capillary dilatation was present within the affected drainage area (white arrowhead) with dye leakage in late frames, resulting in cystoid macular edema (black arrowhead) which was sectorial on macular OCT (optical coherence tomography) (star). Serological tests, after anterior chamber puncture, confirmed local production of specific anti toxoplasma antibodies. The patient was treated with clindamycin 150 mg four times a day and prednisolone 60 mg daily. After 6 weeks of antiparasitic therapy the lesion became inactive and visual acuity improved. Repeat fluorescein angiography and OCT demonstrated resorption of the macular edema.

**Figure 1:**
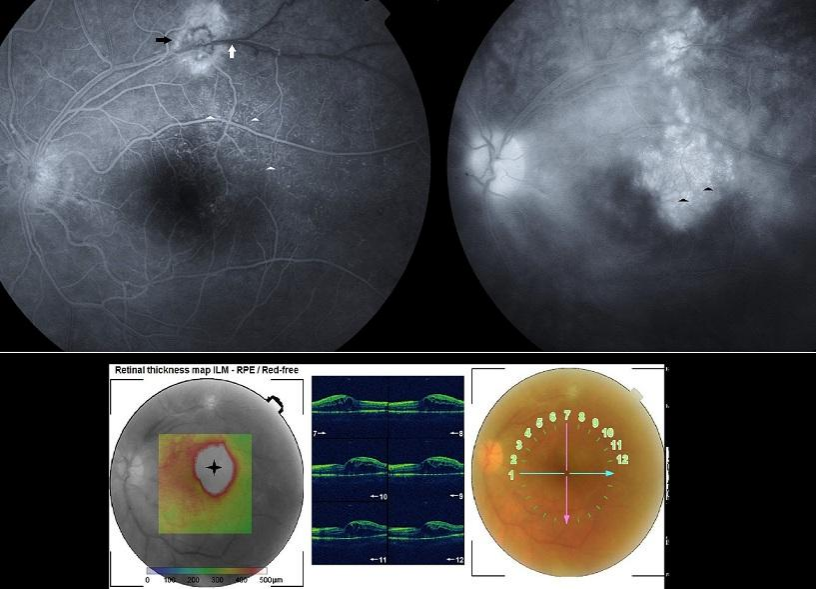
A) delayed filling and tortuosity of the superotemporal vein (white arrow) with capillary dilatation in the affected drainage area (white arrowhead) and cystoid macular edema in the late frames (black arrowhead); B) OCT mapping showing sectorial macular edema (black star)

